# The effects of abaloparatide on hip geometry and biomechanical properties in Japanese osteoporotic patients assessed using DXA-based hip structural analysis: results of the Japanese phase 3 ACTIVE-J trial

**DOI:** 10.1007/s11657-023-01344-5

**Published:** 2023-11-30

**Authors:** Teruki Sone, Kazuhiro Ohnaru, Takumi Sugai, Akiko Yamashita, Nobukazu Okimoto, Tetsuo Inoue, Toshio Matsumoto

**Affiliations:** 1 Department of Nuclear Medicine, Kawasaki Medical School, 577, Matsushima, Kurashiki, Okayama, 701-0192 Japan; 2 Department of Orthopedics, Traumatology & Spine Surgery, Kawasaki Medical School, Okayama, Japan; 3 Division of Pharmaceutical Development and Production, Teijin Pharma Limited, Tokyo, Japan; 4 Okimoto Clinic, Hiroshima, Japan; 5 Aoyama General Hospital, Aichi, Japan; 6 Fujii Memorial Institute of Medical Sciences, Tokushima University, Tokushima, Japan

**Keywords:** Abaloparatide, Bone mineral density, Dual-energy X-ray absorptiometry, Hip geometry, Hip structural analysis, Osteoporosis

## Abstract

**Summary:**

Daily subcutaneous injection of 80 μg abaloparatide increased bone mineral density in Japanese patients with osteoporosis at high fracture risk in the ACTIVE-J trial. Dual-energy X-ray absorptiometry–based hip structural analysis from ACTIVE-J data showed improved hip geometry and biomechanical properties with abaloparatide compared with placebo.

**Purpose:**

Abaloparatide (ABL) increased bone mineral density (BMD) in Japanese patients with osteoporosis at high fracture risk in the ACTIVE-J trial. To evaluate the effect of ABL on hip geometry and biomechanical properties, hip structural analysis (HSA) was performed using ACTIVE-J trial data.

**Methods:**

Hip dual-energy X-ray absorptiometry scans from postmenopausal women and men (ABL, *n* = 128; placebo, *n* = 65) at baseline and up to week 78 were analyzed to extract bone geometric parameters at the narrow neck (NN), intertrochanteric region (IT), and proximal femoral shaft (FS). Computed tomography (CT)-based BMD and HSA indices were compared between baseline and week 78.

**Results:**

ABL treatment showed increased mean percent change from baseline to week 78 in cortical thickness at the NN (5.3%), IT (5.3%), and FS (2.9%); cross-sectional area at the NN (5.0%), IT (5.0%), and FS (2.6%); cross-sectional moment of inertia at the NN (7.6%), IT (5.1%), and FS (2.5%); section modulus at the NN (7.4%), IT (5.4%), and FS (2.4%); and decreased mean percent change in buckling ratio (BR) at the IT (− 5.0%). ABL treatment showed increased mean percent change in total volumetric BMD (vBMD; 2.7%) and trabecular vBMD (3.2%) at the total hip and decreased mean percent change in BR at femoral neck (− 4.1%) at week 78 vs baseline. All the changes noted here were significant vs placebo (*P* < 0.050 using *t*-test).

**Conclusion:**

A 78-week treatment with ABL showed improvement in HSA parameters associated with hip geometry and biomechanical properties vs placebo.

**Trial registration:**

JAPIC CTI-173575

**Supplementary Information:**

The online version contains supplementary material available at 10.1007/s11657-023-01344-5.

## Introduction

Osteoporosis is a progressive bone metabolic disease characterized by compromised bone strength, which leads to increased bone fragility and risk of fractures [1, 2]. The loss or changes in bone density and microarchitecture in osteoporosis are attributed to an imbalance between osteoblastic bone formation and osteoclastic bone resorption, consequently resulting in osteoporotic fractures [2]. Osteoporosis is age-related and affects women more frequently than men, and osteoporotic fractures, especially vertebral and hip fractures, are associated with increased mortality, morbidity, and medical costs and decreased quality of life [3].

Japan has the largest aging population in the world [4, 5]. The number of elderly people at high fracture risk is increasing, with an estimated 193,400 events (149,300 in women and 44,100 in men) of hip fractures in 2017 [4]. Thus, prevention of osteoporosis and related fractures is of high importance in the aging Japanese population. The risk of fractures, estimated using bone mineral density (BMD) as a measure of bone strength, can be prevented by improving bone strength and early assessment before the first events of fracture [6]. Current treatment options to prevent osteoporotic fractures in Japan include bisphosphonates, parathyroid hormone (PTH) derivatives, selective estrogen receptor modulators, vitamin D analogs, and biological agents including denosumab and romosozumab [6–8]. Teriparatide, a recombinant PTH (1-34), is widely used as an anabolic agent for preventing fractures in patients with severe osteoporosis with an imminent fracture risk [9, 10]. However, teriparatide not only promotes bone formation but also stimulates bone resorption [11], which may be associated with increased porosity in the cortical bone [12]. In fact, it was shown that denosumab in combination with teriparatide inhibited teriparatide-induced bone resorption [12, 13]. Abaloparatide (ABL), a synthetic PTH-related peptide analog that selectively binds to the RG conformation of PTH type 1 receptor, demonstrated increased BMD and reduced the risk of osteoporosis-related vertebral and nonvertebral fractures compared with placebo (PBO) or teriparatide among postmenopausal women with osteoporosis in the pivotal ACTIVE phase 3 trial [14] and its extension ACTIVExtend trial [15]. Moreover, ABL showed a more pronounced anabolic effect and lesser bone resorption than teriparatide [14]. In Japanese patients with osteoporosis at high fracture risk, ABL demonstrated a potent increase in BMD in the lumbar spine, total hip, and femoral neck (FN) in the randomized, double-blind, multicenter, PBO-controlled, parallel group, phase 3 ACTIVE-J trial [16].

While dual-energy X-ray absorptiometry (DXA) and quantitative computed tomography (QCT) are common tools for measuring BMD [17], hip structural analysis (HSA), a technique that uses the properties of DXA images to derive geometric parameters for the hip that are associated with bone strength, has been used to assess the effect of anabolic agents on hip geometry [7, 18]. HSA has been demonstrated to be an excellent predictor of proximal femoral fracture risk [19]. Subgroup analysis from the ACTIVE and ACTIVExtend trials using three-dimensional (3D)-DXA demonstrated improvement in total hip volumetric BMD (vBMD) and hip bone strength indices with ABL [20, 21]. It has been shown previously that hip geometry between Japanese and American populations is different [22]; however, the clinical efficacy of ABL, especially on hip bone strength, has not been demonstrated in the Japanese population. Therefore, the current exploratory analysis assessed the efficacy of ABL in improving hip geometry and biomechanical properties evaluated using HSA based on DXA and computed tomography (CT) scans using data from the ACTIVE-J trial.

## Methods

### Study design and participants

ACTIVE-J was a multicenter, randomized, double-blind, parallel group, PBO-controlled, phase 3 trial in which patients were randomized 2:1 to receive 80 μg of ABL (daily subcutaneous self-injection) or PBO for 18 months (78 weeks). The study period spanned from the day of obtaining informed consent to the day of the last visit, defined as the week 78 visit, or the day of withdrawal from the study, or the last day of tests after withdrawal. The study participants, described previously in detail [16], were Japanese postmenopausal women and men aged 55–85 years with osteoporosis at a high risk of fractures and who had lumbar spine BMD data measurable using DXA. All patients in this study fulfilled the inclusion and exclusion criteria of the ACTIVE-J trial. Among the participants in the ACTIVE-J trial, those with evaluable data for DXA or CT at each time point were included in the current analyses.

### Structural analysis

#### DXA-based HSA

BMD was measured at the total hip and narrow neck (NN) with conventional two-dimensional (2D) DXA using the QDR, DELPHI, Explorer, Discovery, and Horizon systems (Hologic 134 Inc., Marlborough, MA, USA), as described earlier [16], at baseline; weeks 12, 24, 48, and 78; and the last visit. To assess the cross-sectional geometric and biomechanical parameters of the proximal femur, the NN (the narrowest diameter of the FN); intertrochanteric region (IT) along the bisector of the neck-shaft angle; and proximal femoral shaft (FS) 2 cm distal to the midpoint of the lesser trochanter were scanned [23]. Hip DXA scans were analyzed for DXA-based HSA using Hologic Apex system software version 5.6 (Madison, WI) in accordance with a standardized HSA protocol [23]. All cross-sectional geometries were calculated from mass profile distributions converted to linear thickness by dividing each pixel value by the effective mineral density of fully mineralized tissue. Geometric parameters and derived strength indices included the periosteal outer diameter (OD; distance between the outer margins of the cross-section), average cortical thickness (CoTh; as a measure of cortical bone width), bone cross-sectional area (CSA; surface of the bone tissue as a measure of resistance to forces directed along the long axis of the bone), cross-sectional moment of inertia (CSMI; index of bending strength), section modulus (SM; index of resistance to bending forces), and buckling ratio (BR; measure of the risk of buckling), as described previously [7, 18, 24].

#### Quantitative computed tomography

To support the improved change in hip structure with ABL, patients in each treatment group were monitored using 3D QCT to assess changes in total vBMD, cortical vBMD, and trabecular vBMD in the total hip and FN. Patients enrolled at sites equipped with 3D QCT analysis systems were eligible for the analysis. Scans were acquired using one of two CT machines, namely, Aquilion (Canon Medical Systems Corp., Tochigi, Japan; slice thickness: 0.5 mm) and MX 16 (Philips and Neusoft Medical Systems Co., Shenyang, China; slice thickness, 1.0 mm). All scans were performed at a tube voltage of 120–140 kVp and tube current of 250 mA (Online Resource 1). CT equipment and scanning conditions were standardized at baseline and week 78 after the administration of ABL or PBO. CT values were converted to vBMD using QCT Pro™ calibration phantom (Mindways Software Inc. Austin, TX). CT scans of patients were excluded from the analysis when any of the following exclusion criteria were met: (1) improper body positioning, (2) movement of the body, (3) foreign bodies in the image, (4) images of a different side of the body from baseline observation, (5) change in analysis equipment or scan modes, or (6) other reasons judged reasonable by the investigator.

#### CT-based HSA

QCT data were utilized for the analysis of proximal femoral geometry by CT-based HSA using QCT Pro Software version 5.1.3 and QCT Pro Bone Investigational Toolkit (BIT version 2.0, Mindways Software Inc. Austin, TX). Data were evaluated using the CTXA Hip Exam Analysis protocol (Mindways Software Inc. Austin, TX), followed by QCT BIT processing according to the standard procedure to obtain HSA indices, as described previously [25, 26].

A series analysis was performed for the FN, and the HSA indices (average CoTh, maximum CSMI, maximum SM, and BR) were obtained from 11 slices.

### Outcomes

The outcomes assessed were DXA-based HSA indices for the NN, IT, and FS at baseline (before treatment initiation); weeks 12, 24, 48, and 78; and the last visit, and CT-based HSA indices at baseline and week 78 for the ABL and PBO groups. The mean percent changes from baseline for each parameter were also compared between the treatment groups.

### Statistical analysis

The full analysis set (FAS) included all randomized patients who received ≥ 1 dose of ABL or PBO and had a baseline and ≥ 1 post-baseline scan. Statistical analysis was conducted on the HSA indices obtained from DXA of 193 evaluable patients (ABL, 128; PBO, 65) and from CT of 70 evaluable patients (ABL, 47; PBO, 23). Results for postmenopausal women and men were pooled because the number of men in each group was small. Descriptive statistics were used to calculate DXA-based or CT-based HSA indices at each evaluation time point in each treatment group and are shown as mean ± standard deviation (SD). Mean percent change, along with the 95% confidence interval (CI), in HSA indices from baseline at each evaluation time point in the treatment groups and between-group differences were calculated. The mean percent change from baseline in each parameter was compared between the ABL and PBO groups at each time point using a *t*-test at a two-sided significance level of 0.050. No missing data were imputed in the analysis. Statistical analysis was performed using SAS version 9.3 (SAS Inc., Cary, NC, USA).

## Results

### Baseline characteristics

In the ACTIVE-J trial, 140 and 72 patients received ABL and PBO, respectively, and 136 (122 postmenopausal women, 14 men) and 70 (64 postmenopausal women, 6 men) patients, respectively, were included in the FAS for primary outcome analysis [16]. The overall baseline characteristics in the FAS were similar between treatment groups (mean age, 68.6 vs 68.8 years; mean total hip T-score, − 2.3 vs − 2.3; mean FN T-score, − 2.8 vs − 2.8; ≥ 1 prevalent vertebral fracture, 44.9% vs 27.1% in the ABL vs PBO group, respectively) [16]. Among these patients, 128 (114 postmenopausal women, 14 men) from the ABL group and 65 (60 postmenopausal women, 5 men) from the PBO group were included in the DXA-based HSA, and 47 patients (42 postmenopausal women, 5 men) from the ABL group and 23 (19 postmenopausal women, 4 men) from the PBO group were included in the CT-based HSA. Table 1 shows the bone geometric parameters of patients at baseline assessed using DXA-based HSA. The parameters were similar in both groups.

**Table 1:** Bone geometric parameters (DXA-based HSA indices) at baseline

Variables	NN		IT		FS	
	PBO	ABL	PBO	ABL	PBO	ABL
*n*, total	65	128	65	128	65	128
*n*, men	5	14	5	14	5	14
Parameters (unit), mean ± SD						
OD (cm)	3.24 ± 0.36	3.23 ± 0.30	5.46 ± 0.37	5.46 ± 0.44	2.86 ± 0.23	2.89 ± 0.21
CoTh (cm)	0.12 ± 0.02	0.12 ± 0.02	0.28 ± 0.04	0.28 ± 0.05	0.40 ± 0.06	0.40 ± 0.07
CSA (cm^2^)	1.98 ± 0.21	1.97 ± 0.31	3.39 ± 0.44	3.40 ± 0.59	3.09 ± 0.39	3.12 ± 0.47
CSMI (cm^4^)	1.56 ± 0.42	1.56 ± 0.48	8.88 ± 2.09	9.12 ± 2.61	2.45 ± 0.58	2.54 ± 0.66
SM (cm^3^)	0.85 ± 0.18	0.86 ± 0.21	2.81 ± 0.55	2.86 ± 0.65	1.64 ± 0.28	1.68 ± 0.32
BR	15.26 ± 3.65	15.30 ± 3.58	11.48 ± 1.93	11.73 ± 2.06	3.76 ± 0.73	3.85 ± 0.83

Among the participants in the ACTIVE-J trial [16], those with evaluable data for DXA were included in the current analysis. Mean and SD values have been rounded off to two decimal points

*ABL* abaloparatide, *BR* buckling ratio, *CoTh* cortical thickness, *CSA* cross-sectional area, *CSMI* cross-sectional moment of inertia, *DXA* dual-energy X-ray absorptiometry, *FS* femoral shaft, *HSA* hip structural analysis, *IT* intertrochanteric region, *NN* narrow neck, *OD* outer diameter, *PBO* placebo, *SD* standard deviation, *SM* section modulus

### Effects of ABL on bone geometry and biomechanical parameters: DXA-based HSA indices

The ACTIVE-J trial demonstrated that the BMD of the total hip and FN increased significantly after 78 weeks of ABL injection [16]. ABL increased the mean BMD of the total hip by 4.4% and of the FN by 4.8% from baseline to week 78, whereas the mean BMD did not change from baseline with PBO. We further assessed the effect of ABL on bone structural parameters using DXA-based HSA.

#### Effect of ABL on bone geometry indices

**Fig. 1:**
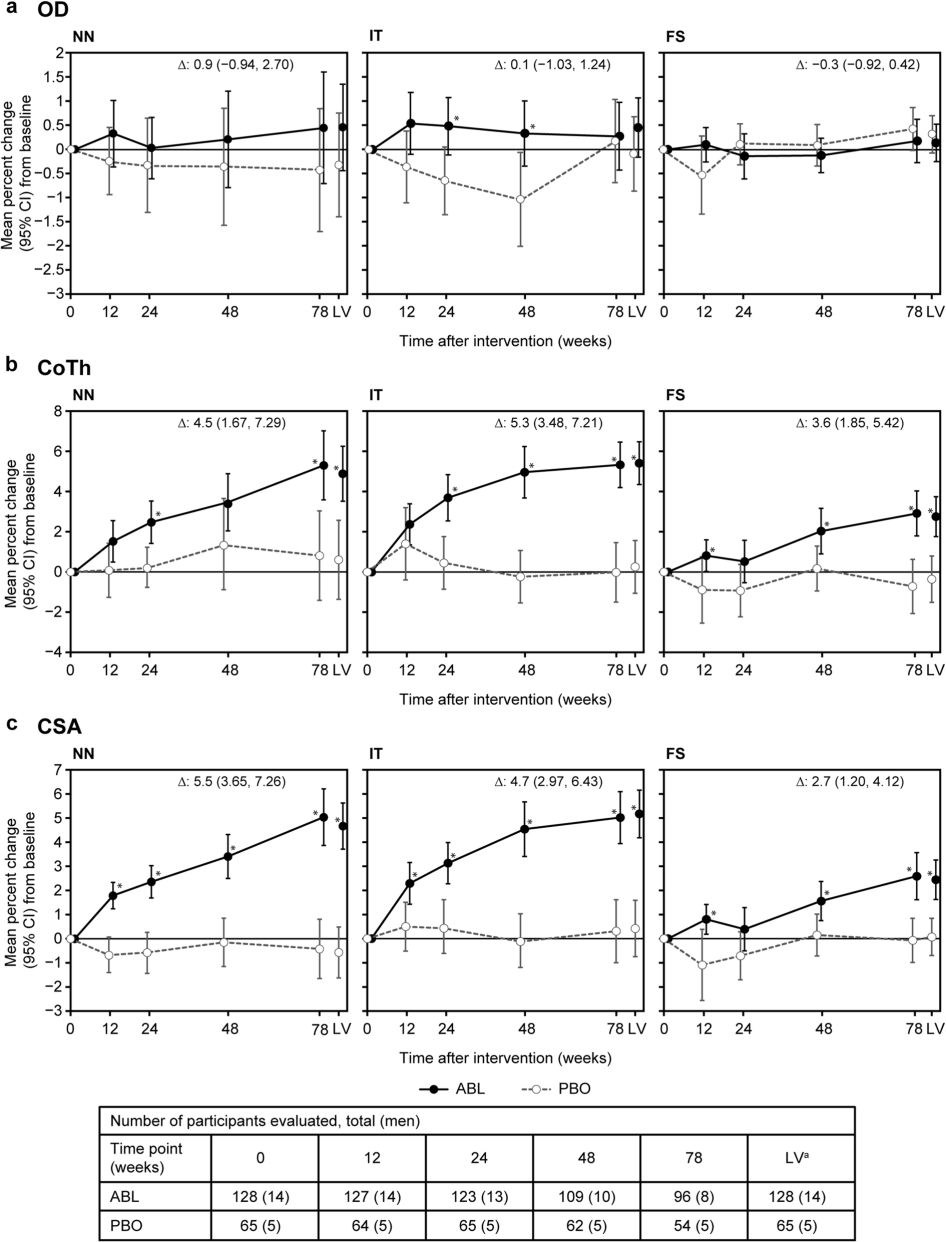
Mean percent change from baseline in DXA-based bone geometry HSA indices. (**a**) OD, (**b**) CoTh, and (**c**) CSA. Data labels in each panel indicate the mean percent difference (95% CI) between ABL and PBO at 78 weeks. *P < 0.050 (vs PBO) based on a *t*-test. ^a^LV, defined as week 78 visit or day of withdrawal from the study; Δ, mean difference; ABL, abaloparatide; CI, confidence interval; CoTh, cortical thickness; CSA, cross-sectional area; DXA, dual-energy X-ray absorptiometry; FS, femoral shaft; HSA, hip structural analysis; IT, intertrochanteric region; LV, last visit; NN, narrow neck; OD, outer diameter; PBO, placebo

Comparisons of bone geometry indices (OD, CoTh, and CSA) from baseline between the ABL and PBO groups over time are shown in Fig. 1. Compared with that at baseline, ABL increased the CoTh and CSA over time at all assessment sites. At week 78, the ABL group showed an increased mean percent change from baseline in CoTh at the NN (5.3%), IT (5.3%), and FS (2.9%) and in CSA at the NN (5.0%), IT (5.0%), and FS (2.6%). In the PBO group, the mean percent change from baseline to week 78 in the OD, CoTh, and CSA was within the range of − 0.7 to 0.8%.

Compared with PBO, 78 weeks of ABL injection showed a significant difference in CoTh at the NN (4.5%; 95% CI, 1.67, 7.29), IT (5.3%; 95% CI, 3.48, 7.21), and FS (3.6%; 95% CI, 1.85, 5.42), and in CSA at the NN (5.5%; 95% CI, 3.65, 7.26), IT (4.7%; 95% CI, 2.97, 6.43), and FS (2.7%; 95% CI, 1.20, 4.12).

#### Effect of ABL on bone strength indices

**Fig. 2:**
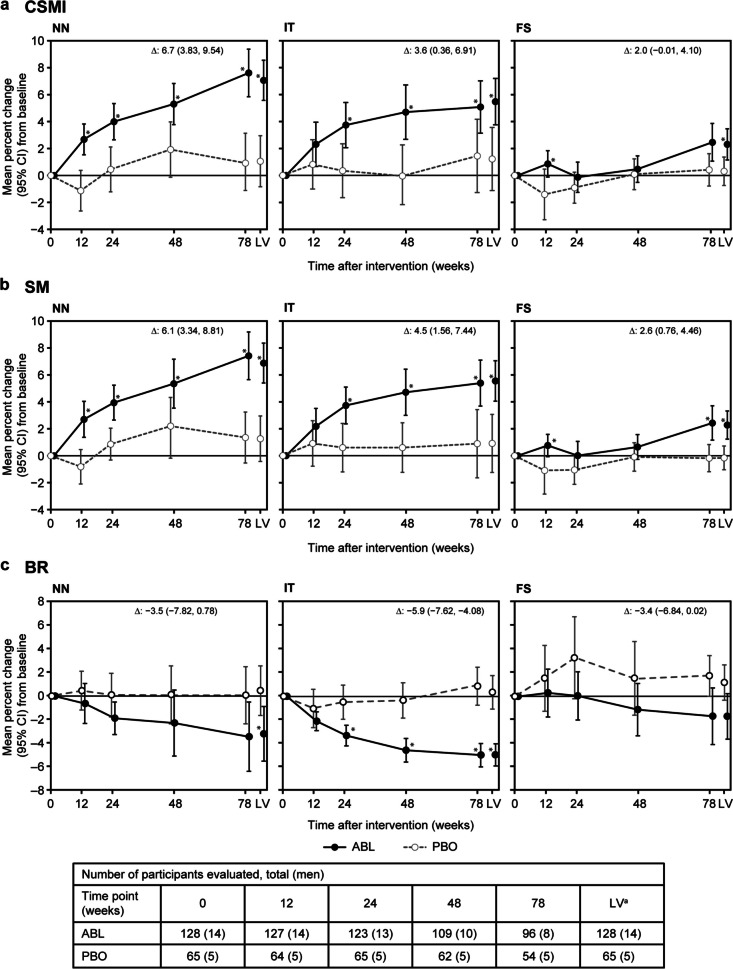
Mean percent changes from baseline in DXA-based bone strength HSA indices. (**a**) CSMI, (**b**) SM, and (**c**) BR. Data labels in each panel indicate the mean percent difference (95% CI) between ABL and PBO at 78 weeks. *P < 0.050 (vs PBO) based on a *t*-test. ^a^LV, defined as week 78 visit or day of withdrawal from the study; Δ, mean difference; ABL, abaloparatide; BR, buckling ratio; CI, confidence interval; CSMI, cross-sectional moment of inertia; DXA, dual-energy X-ray absorptiometry; FS, femoral shaft; HSA, hip structural analysis; IT, intertrochanteric region; LV, last visit; NN, narrow neck; PBO, placebo; SM, section modulus

Comparisons of bone strength indices (CSMI, SM, and BR) from baseline between the ABL and PBO groups over time are shown in Fig. 2. Compared with that at baseline, ABL increased the CSMI and SM over time at the NN and IT, and from week 48 at the FS. At week 78, the ABL group showed an increased mean percent change from baseline in CSMI at the NN (7.6%), IT (5.1%), and FS (2.5%) and in SM at the NN (7.4%), IT (5.4%), and FS (2.4%). The mean percent change from baseline to week 78 in CSMI and SM in the PBO group was within the range of − 0.2 to 1.5%. The ABL group showed improvements in mean percent change from baseline to week 78 in BR at the NN (− 3.5%) and IT (− 5.0%). In the PBO group, the mean percent change from baseline to week 78 in BR was within the range of 0.1 to 1.7%.

Compared with PBO, 78 weeks of ABL injection showed a significant difference in CSMI at the NN (6.7%; 95% CI, 3.83, 9.54) and IT (3.6%; 95% CI, 0.36, 6.91); in SM at the NN (6.1%; 95% CI, 3.34, 8.81), IT (4.5%; 95% CI, 1.56, 7.44), and FS (2.6%; 95% CI, 0.76, 4.46); and in BR at the IT (− 5.9%; 95% CI, − 7.62, − 4.08).

### CT-based analysis

**Fig. 3:**
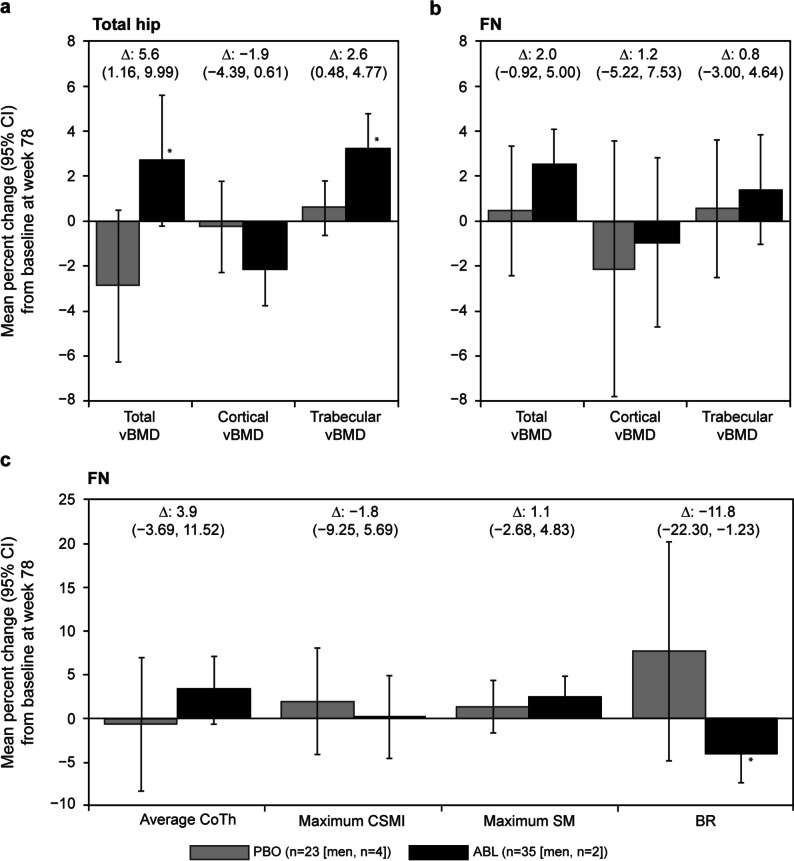
CT-based vBMD and HSA. (**a**) Total hip vBMD, (**b**) FN vBMD, and (**c**) HSA at FN. Data labels in each panel indicate the mean percent difference (95% CI) between the ABL (*n* = 35) and PBO (*n* = 23) groups at 78 weeks. *P < 0.050 (vs PBO) based on a *t*-test. Δ, mean difference; ABL, abaloparatide; BMD, bone mineral density; BR, buckling ratio; CI, confidence interval; CoTh, cortical thickness; CSMI, cross-sectional moment of inertia; CT, computed tomography; HSA, hip structural analysis; FN, femoral neck; PBO, placebo; SM, section modulus; vBMD, volumetric BMD

Changes from baseline to week 78 in CT-based vBMD and CT-based HSA parameters are shown in Fig. 3. At week 78, 35 patients (33 postmenopausal women, 2 men) from the ABL group and 23 (19 postmenopausal women, 4 men) from the PBO group were included in the CT-based HSA. For both the total hip and FN, the ABL group showed an increased mean percent change from baseline to week 78 in total vBMD (total hip, 2.7%; FN, 2.5%) and trabecular vBMD (total hip, 3.2%; FN, 1.4%) and a decrease in the mean percent change from baseline in cortical vBMD (total hip, − 2.1%; FN, − 1.0%). The mean percent change from baseline at week 78 in the PBO group was within the range of − 2.9 to 0.5% for total vBMD, 0.6% for trabecular vBMD, and − 2.1 to − 0.3% for cortical vBMD. Compared with PBO, 78 weeks of ABL injection resulted in a significant increase in the mean difference in total vBMD (5.6%; 95% CI, 1.16, 9.99) and trabecular vBMD (2.6%; 95% CI, 0.48, 4.77) at the total hip (Fig. 3a and b).

In the ABL group, BR showed a significant improvement vs PBO in the mean percent change from baseline to week 78 at the FN (mean percent change, − 4.1% vs 7.7%; mean difference, − 11.8%; 95% CI, − 22.30, − 1.23). No significant difference vs PBO was observed in the mean percent change from baseline in average CoTh, maximum CSMI, and maximum SM at the FN in the ABL group (Fig. 3c).

## Discussion

This exploratory analysis of the ACTIVE-J trial demonstrated the changes in bone geometry and biomechanical properties at the proximal femur by using conventional DXA-based HSA with daily injection of 80 μg ABL for 78 weeks. To the best of our knowledge, this is the first longitudinal study to assess the effect of ABL on bone strength indices in elderly Japanese patients with osteoporosis at high risk of fracture. Our results indicate time-dependent potential improvement in bone strength based on HSA indices (CSMI, SM, and BR) at the NN, IT, and FS.

After 78 weeks of ABL treatment, the total hip and FN BMD increased from baseline when evaluated using conventional DXA [16]. This was supported by our QCT analysis, which showed an increase in vBMD in the total and trabecular region of the FN with ABL. Our results suggest that ABL improved trabecular bone in the total hip. The decreasing trend in cortical vBMD was comparable between the ABL and PBO groups at the FN.

At the NN and IT, hip geometry indices of DXA-based HSA, such as CSA, improved from week 12 of ABL treatment, whereas CoTh improved from week 24 compared with PBO. At the FS, CoTh and CSA also improved after 12 weeks of ABL treatment. Moreover, improvement in CoTh and CSA was greater in the ABL vs PBO group at week 78 at all assessed regions. In contrast, the OD remained unchanged from baseline to 78 weeks of ABL treatment in all investigated regions. At the NN and IT, although the mean percent change in OD did not differ significantly, the OD value was higher in the ABL treatment group than in the PBO group. There is no clear report that suggests that the OD of the FN increases with teriparatide treatment. However, recently, it has been reported that ABL has a stronger modeling-based bone formation–promoting effect at the periosteum compared with teriparatide [27], which may have contributed to a higher OD value than that of PBO in this study. Additionally, the lower baseline BMD and thinner cortical bones of the FN and total hip in ACTIVE-J participants receiving ABL (mean T-score [SD], − 2.8 [0.7] and − 2.3 [0.7], respectively) compared with those in the ACTIVE study (mean T-score [SD], − 2.2 [0.6] and − 1.9 [0.7], respectively) may increase error variability and make evaluation of changes in OD difficult even with QCT [15, 16, 28]. Several studies have shown improvement in hip geometry with daily or weekly teriparatide in patients with osteoporosis by using DXA-based HSA [29–31]. Hip geometry changes with a weekly injection of teriparatide for 72 weeks showed almost the same pattern of geometry indices as that in the present study [30]. However, an improving tendency in bone geometry parameters from the initial stages (weeks 12 and 24) of ABL treatment suggests an early effect of ABL.

Improvements in hip strength indices, such as CSMI and SM, were observed at the NN from week 12 and at the IT from week 24 of ABL treatment compared with PBO, indicating a rapid effect of ABL on bone strength. At the FS, the SM improved after 78 weeks of ABL treatment vs PBO. Improvement (decrease) in BR was observed at the IT from week 24 of ABL treatment. Moreover, improvement in CSMI at the NN and IT; SM at the NN, IT, and FS; and BR at the IT was greater in the ABL vs PBO group at week 78. As BR is predicted to explain structural strength with low bone mass, a greater decrease in BR at the IT shows greater strength against compressive buckling loads. Interestingly, similar results were seen in Japanese patients in whom denosumab improved several geometric parameters calculated using HSA for 3 years [7]. Compared with PBO, denosumab significantly improved CoTh, CSA, CSMI, SM, and BR in the NN, IT, and FS [7]. Our results were also in accordance with 3D-DXA analyses of hip DXA scans from a subgroup of participants in the ACTIVE trial who showed greater improvements in CSMI and SM of the FN and lower shaft after 6 and 18 months with ABL vs teriparatide treatment [20]. These results suggest that daily ABL injection improves deterioration in bone structural strength with increasing CoTh, CSA, and CSMI, without changes in the OD in all the investigated regions. Therefore, these findings indicate the potential of ABL in reducing the risk of hip fracture. As reported previously, in the ACTIVE-J trial, new vertebral fractures occurred in 4 vertebrae of 3 patients (4.3%) in the PBO group, whereas no vertebral fractures were observed in patients in the ABL group (absolute risk reduction, − 4.3%). New nonvertebral fractures occurred in 2 (2.9%) and 3 (2.2%) patients in the PBO and ABL groups, respectively [16]. However, owing to the limited number of patients who experienced nonvertebral hip fractures, further analyses of the fracture events and hip parameters were not feasible.

In this exploratory analysis, male and female hip geometry data were pooled and analyzed. The ACTIVE-J trial reported that the effect of ABL on increasing lumbar spine BMD was almost the same in the analysis results when postmenopausal women and men were pooled, as well as in the analysis of postmenopausal women alone [16]. The hip geometry might be different and changes in men may differ from those observed in postmenopausal women. However, most HSA parameters are heavily influenced by BMD to an extent that those parameters would be expected to have similar patterns in men and postmenopausal women in this study. This is in congruence with a previous study that analyzed the effect of denosumab on HSA parameters in a pooled cohort of Japanese men and postmenopausal women with osteoporosis [7].

This study has some limitations. The major limitations are the small number of patients in the ACTIVE-J trial and the lack of statistical tests to compare bone parameters at baseline and those at each time point. Second, the least significant change (LSC) was not available in the current study, and thus, discussion regarding LSC values based on the published literature was not feasible. Third, the number of patients who had CT scans available for analysis was limited. Moreover, CT imaging was performed using two different models that had two different slice thicknesses, which might have led to low resolution and higher variability and, in turn, resulted in insufficient support for the 2D-HSA data. The current HSA technique is limited by its 2D nature. Nevertheless, our results with 2D-DXA are similar to the results from the ACTIVE and ACTIVExtend trials, which used 3D-DXA analysis [20, 21].

In conclusion, a 78-week treatment with ABL showed improvement in HSA parameters vs PBO in Japanese patients with osteoporosis at high fracture risk. The results suggest the efficacy of daily subcutaneous injection of ABL 80 μg in improving HSA parameters associated with hip geometry and biomechanical properties.

### Supplementary information

ESM 1 (PDF 14 kb)

## Data Availability

Restrictions apply to the availability of some or all data generated or analyzed during this study to preserve patient confidentiality or because they were used under license.
