# Supplementary material for: The effects of abaloparatide on hip geometry and biomechanical properties in Japanese osteoporotic patients assessed using DXA-based hip structural analysis: results of the Japanese phase 3 ACTIVE-J trial
Source: Arch Osteoporos. 2023 Nov 30;18(1):146. doi: 10.1007/s11657-023-01344-5 (PMC10687120; doi:10.1007/s11657-023-01344-5)
Supplement: Supplementary file 1 — (PDF 14 kb) [file 11657_2023_1344_MOESM1_ESM.pdf]

Supplementary Information

Title:

The effects of abaloparatide on hip geometry and biomechanical properties in Japanese osteoporotic patients assessed using DXA-based hip structural analysis: results of the Japanese phase 3 ACTIVE-J trial

Journal name: *Archives of Osteoporosis*

Authors:
Teruki Sone,[1] Kazuhiro Ohnaru,[2] Takumi Sugai,[3] Akiko Yamashita,[3] Nobukazu Okimoto,[4] Tetsuo Inoue,[5] Toshio Matsumoto[6]

Affiliations:
[1]Department of Nuclear Medicine, Kawasaki Medical School, Okayama, Japan
[2]Department of Orthopedics, Traumatology & Spine Surgery, Kawasaki Medical School, Okayama, Japan
[3]Division of Pharmaceutical Development and Production, Teijin Pharma Limited, Tokyo, Japan
[4]Okimoto Clinic, Hiroshima, Japan
[5]Aoyama General Hospital, Aichi, Japan
[6]Fujii Memorial Institute of Medical Sciences, Tokushima University, Tokushima, Japan

Corresponding author:
Teruki Sone, MD
Department of Nuclear Medicine, Kawasaki Medical School, 577, Matsushima, Kurashiki 701-0192, Okayama Japan
E-mail: tsone@med.kawasaki-m.ac.jp

Online Resource 1 CT scan parameters

Parameter	Setting
Beam pitch	Approximately 0.5 to 1
Slice thickness	0.5 mm for Aquilion (Canon Medical Systems Corp., Tochigi, Japan), 1.0 mm for MX 16 (Philips and Neusoft Medical Systems Co., Shenyang, China)
Pixel size	0.6 to 0.7 mm, with a SFOV of large or DFOV of 38 cm
Matrix size for image reconstruction	512 × 512
Tube voltage	120 to 140 kVp (fixed)
Tube current	250 mA (fixed)

CT, computed tomography; DFOV, display field of view; SFOV, scan field of view
